# Changes in Muscle Mass after Botulinum Toxin Injection in Children with Spastic Hemiplegic Cerebral Palsy

**DOI:** 10.3390/toxins13040278

**Published:** 2021-04-14

**Authors:** Dongwoo Lee, Jaewon Kim, Ja-Young Oh, Mi-Hyang Han, Da-Ye Kim, Ji-Hye Kang, Dae-Hyun Jang

**Affiliations:** Department of Rehabilitation Medicine, Incheon St. Mary’s Hospital, College of Medicine, The Catholic University of Korea, Seoul 06591, Korea; violetfear1@naver.com (D.L.); jw2356@naver.com (J.K.); arcayje@hanmail.net (J.-Y.O.); gold830@naver.com (M.-H.H.); mnikdy@gmail.com (D.-Y.K.); rkdwlgp91@naver.com (J.-H.K.)

**Keywords:** cerebral palsy, botulinum toxins, muscular atrophy, spasticity, muscle mass

## Abstract

We aimed to evaluate muscle mass changes after injection of botulinum toxin (BoNT) in children with spastic hemiplegic cerebral palsy (CP). Children aged between 2 and 12 years who were diagnosed with hemiplegic CP with spastic equinus foot were prospectively recruited and administered BoNT in the affected leg. Lean body mass (LBM) of both legs and total limbs was measured by dual-energy X-ray absorptiometry (DXA) preinjection and 4 and 12 weeks after injection. A total of 15 children were enrolled into the study. LBM of both legs and total limbs increased significantly over 12 weeks of growth. The ratio of LBM of the affected leg to total limbs and to the unaffected leg significantly reduced at 4 weeks after injection compared with preinjection but significantly increased at 12 weeks after injection compared with 4 weeks after injection. In conclusion, the muscle mass of the affected leg after BoNT injection in children with hemiplegic spastic CP decreased at 4 weeks after BoNT injection but significantly recovered after 12 weeks after injection.

## 1. Introduction

Cerebral palsy (CP) is a nonprogressive neurodevelopmental disorder that can cause physical disability, such as delayed motor development, gait disturbance, or activity limitation [1,2]. CP affects approximately 2 neonates in every 1000 live births and is the most common cause of serious physical disability in childhood [3,4]. Approximately 60–85% of patients with CP are classified as the spastic type [5]. Spasticity contributes to the shortening of muscles as well as secondary muscle contracture, torsion of the limb bones, and instability of the joint, which worsen the functional movement of the child [6]. Botulinum toxin (BoNT) type A injections are widely used to manage spasticity, pain, and functional outcomes in CP and have proved to be effective in improving change in equinus foot and gait parameters [7]. Although it is generally considered safe, there is a concern that BoNT injection may negatively affect muscle mass and strength [7,8,9,10]. In studies conducted to investigate the histopathologic changes in muscle fibers after BoNT type A injection in humans, there was widespread neurogenic atrophy and reduced size of type I and type II fibers [11,12,13]. Schroeder et al. reported a reduction in the cross-sectional area of the gastrocnemius muscle measured 1 year after BoNT injection in two healthy adults [13]. In addition, in an experiment on rats, the BoNT easily passed through muscle fascia, causing weakness of the nontargeted adjacent muscle even at small dosage [14,15]. These findings suggest that BoNT injection can cause muscle atrophy and paralysis in the targeted muscles and the adjacent muscles. On the other hand, it is reported that synergistic muscle hypertrophy occurs after BoNT injection, which is caused by compensatory mechanisms to adapt muscle weakness [16,17]. Therefore, the effect of BoNT injection on the entire leg is still controversial and it has not been presented. Additionally, gait is a process that requires dynamic coordination of both legs, and the effects of BoNT injection on the contralateral leg and functional aspects have not been clarified.

To date, magnetic resonance imaging (MRI) is considered the most precise and reliable quantitative method for measuring total body and regional skeletal muscle mass [18,19]. However, MRI has a disadvantage in that children require sedation as scanning takes a long time. In addition, an MRI scan for the measurement of skeletal muscle mass is practically difficult to perform due to its high cost. As an alternative to MRI, dual-energy X-ray absorptiometry (DXA) is widely used to analyze total and regional appendicular lean soft tissue masses. DXA presents the lean soft tissue mass—i.e., soft tissue mass excluding bone and fat, and including the skeletal muscle—which constitutes the largest fraction of the extremities, blood, vessels, skin, connective tissue, and fat-free adipose tissue. Hence, DXA has a tendency to overestimate the skeletal muscle mass compared with the skeletal volume measured by MRI [20,21]. Nevertheless, previous studies demonstrated that appendicular lean body mass (LBM) measured by DXA correlated well with skeletal muscle mass measured by MRI and can be used to predict appendicular skeletal muscle mass and that DXA is a good indicator of skeletal muscle mass with high precision and accuracy, even in children and adolescents [22,23,24,25,26].

The purpose of this study was to find clues for determining whether BoNT injection in targeted muscles may result in a generalized reduction in the muscle mass of the affected limb, including the surrounding muscles of the targeted muscles, or an increase in the muscle mass of the affected side by synergic muscle hypertrophy or the unaffected side by the effect of compensatory action and functional improvement. In addition, we aimed to investigate the change in skeletal muscle mass after BoNT injection over time. We measured the change in entire LBM of the affected leg, unaffected leg, and total limbs using DXA preinjection as well as at 4 and 12 weeks after injection in children with spastic hemiplegic CP.

## 2. Results

### 2.1. Study Participants

A total of 15 children with spastic hemiplegic CP were enrolled. The baseline characteristics of the children are presented in Table 1. Their mean age was 5.27 (±2.52) years (range, 2–11 years). Time from previous BoNT injection ranges from 7 month to 4 years (median, 11 month). All participants received BoNT unilaterally to the medial and/or lateral head of gastrocnemius muscles (3–8 U/kg). Eight participants also received BoNT to the unilateral tibialis posterior muscle (2–3 U/kg). Other injected muscles include the soleus (one patient; 2 U/kg) and the peroneus longus (two patients; 2–3 U/kg) muscles.

### 2.2. LBM

Comparing the affected and unaffected legs at each period, we found that LBM of the affected side was less than that on the unaffected side, and the difference was statistically significant (2018 vs. 2220 g (90.9%), 1981 vs. 2288 g (86.8%), and 2154 vs. 2398 g (89.9%), respectively, *p* < 0.05). Four weeks after injection, LBM of the affected leg decreased (−1.8%), but the decrease was not statistically significant (*p* = 0.316); however, 12 weeks after injection, LBM was significantly increased compared to preinjection (6.7%, *p* = 0.003) and 4 weeks after injection (8.7%, *p* = 0.008). LBM of the unaffected leg increased significantly at 4 and 12 weeks after injection compared with the preinjection measurement (3.1% (*p* = 0.001) and 8.0% (*p* < 0.001), respectively). LBM of the upper arms increased significantly 12 weeks after injection compared with preinjection and 4 weeks after injection (6.8% (*p* = 0.001) and 5.2% (*p* = 0.009), respectively). LBM of the total limbs also increased after 12 weeks compared with preinjection and 4 weeks after injection (7.3% (*p* < 0.001) and 6.4% (*p* = 0.007), respectively) (Table 2). 

### 2.3. Ratio of LBM

**Figure 1:**
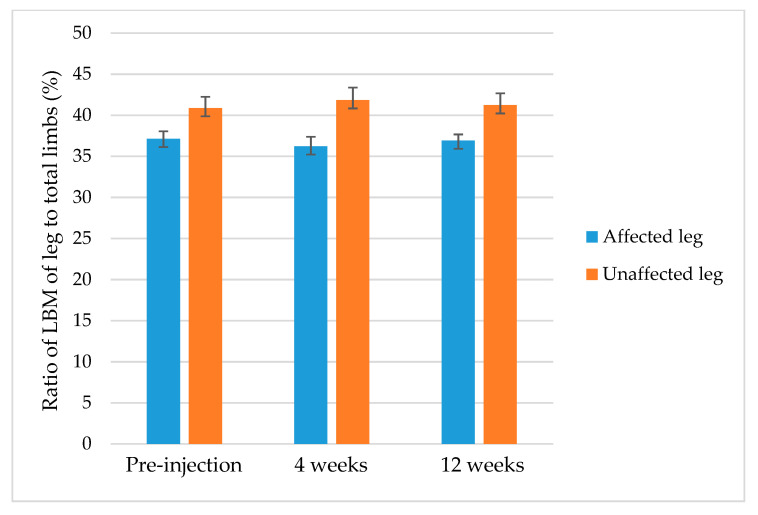
Changes in the mean ratio of lean body mass (LBM) of the affected and the unaffected leg to total limbs after botulinum toxin injection.

The ratio of LBM of the affected leg to total limbs significantly decreased at 4 weeks compared with preinjection (from 37.14% to 36.22%, *p* = 0.001) but significantly increased at 12 weeks compared with 4 weeks after injection (from 36.22% to 36.91%, *p* = 0.001). The ratio of LBM of the unaffected leg to total limbs increased significantly at 4 weeks after injection (from 40.88% to 41.83%, *p* = 0.001). No significant difference was found in the ratios of affected and unaffected leg to total limbs between before and 12 weeks after injection (Table 3, Figure 1). The ratio of the affected leg to the unaffected leg decreased significantly at 4 weeks after injection compared with preinjection (from 90.9% to 86.8%, *p* < 0.001) but increased significantly at 12 weeks compared with 4 weeks after injection (from 86.8% to 89.8%, *p* = 0.013). There was no significant difference between preinjection and 12 weeks after injection (90.9% vs. 89.8%, *p* = 0.151) (Table 4).

## 3. Discussion

The present study is the first to analyze changes in the muscle mass of children with spastic hemiplegic CP after injection of BoNT using DXA. We compared LBM of the affected side with the unaffected side to minimize the bias caused by growth, and the ratio of LBM after injection to preinjection was analyzed to minimize the bias due to individual differences in growth.

As a result, LBM of the affected leg decreased at 4 weeks after injection but not significantly compared with that before injection and significantly increased at 12 weeks after injection compared with before and 4 weeks after injection. The normal growth could partially offset the decrease in LBM of the affected leg at 4 weeks after injection, which was a nonsignificant reduction. LBM ratio of the affected leg to the total limbs and unaffected leg decreased significantly at 4 weeks compared with the ratio before injection. This result suggests that there is a significant reduction in muscle mass over the short term after BoNT injection. However, there was a significant increase at 12 weeks after injection compared with 4 weeks after injection, which means that after a certain period, the loss of muscle mass of the affected leg was restored.

Meanwhile, LBM of the unaffected leg significantly increased before, at 4 weeks, and at 12 weeks after injection. No significant increase in LBM of upper limbs was observed after 4 weeks, but the significant increase in LBM of the unaffected leg and the ratio of the unaffected leg to the total limbs at 4 weeks after injection compared with before injection suggest the possibility that the muscle mass increased as the load on the unaffected leg increased as a compensatory action of the affected leg’s weakness. At 12 weeks, the ratio of LBM of the unaffected leg to the total limbs showed no significant difference compared to that before injection. This suggests a possibility that the compensatory change decreased owing to an increase in the synergic muscle mass of the affected leg by the functional improvement.

No significant difference in LBM of the total limbs was observed for approximately 4 weeks (from preinjection to 4 weeks after injection), but a significant increase was observed in the measurements at intervals of ≥8 weeks (from preinjection and 4 weeks after injection to 12 weeks after injection), which is believed to be a point to be noted in the studies of muscle mass in growing children. In future studies of children with an observation period of over 2 months, consideration of the physiological growth of children is important for interpreting the results.

In addition, the affected leg had less LBM by 10% than the unaffected leg even before injection. This is thought to be related to the fact that 10 of the 15 children had previously undergone BoNT treatment, and that physiologically, children with CP show less leg muscle volume than typically developing children [27]. In addition, muscle deformity may be related to impaired muscle growth and altered adaptation [28].

Only a few studies of changes in muscle volume after BoNT injection in children with CP have been reported until now. There have been two studies on the muscle volume reducing effect of BoNT in children with CP, suggesting that total muscle volume may decrease in the short term after injection but eventually recovers in the long term. These studies analyzed muscle volume using MRI and compared it with the standardized muscle volume according to height and leg length to express the physiological growth of children [16,17]. Although we did not measure the mass of every individual muscle in the present study, LBM of the affected leg recovered at 12 weeks after injection. There was no significant difference in LBM ratio of the affected leg to the unaffected leg between preinjection and 12 weeks after BoNT injection (89.8% vs. 90.9%, *p* = 0.151). This result is consistent with the results of the two previous studies [16,17].

In terms of treatment, the positive effects of BoNT injection on the functional aspects of CP-induced equinus have been reported in several previous studies [7,29]. The functional enhancement after BoNT injection on the gastrocnemius muscle leads to an efficient use of synergistic muscles, such as the soleus, and it causes muscle hypertrophy, strengthening, and reinforcements in muscle mass. By contrast, recovery of the affected leg muscle mass and strength with decreased spasticity leads to functional improvement. We expect that BoNT injection would bring a positive feedback loop between the recovery of muscle mass and functional improvement, and to obtain these positive results, appropriate muscle targeting, dosage, and frequency of dosing tailored to each patient may be required.

This study has several limitations. First, the baseline characteristics of the participants, such as age, body weight, targeted muscle, amount of dose injected, and history of previous injections were heterogeneous. This is due to the inability to establish a homogeneous group of patients who could be followed up with DXA for up to 12 weeks among children with spastic hemiplegic CP who received BoNT injection. Nevertheless, we tried to solve this part through comparison for each child by applying the ratio of the unaffected leg and the affected leg. Studies that include more children are expected to be conducted in the future. Second, in children with CP, muscle deformity may be related to impaired muscle growth and altered adaptation [28]. Therefore, growth rates of the affected and unaffected legs may be different in children with hemiplegic CP. This can lead to underestimation of the ratio of LBM on the affected side to that on the unaffected side, which may have increased the reduction in muscle mass 4 weeks after injection and reduced recovery 12 weeks after injection. Future research to determine whether a significant difference in muscle mass exists between the affected and unaffected sides, according to growth in children who did not receive BoNT injectionn, is expected to provide an answer. Third, we included only children with hemiplegic CP of Gross Motor Function Classification System (GMFCS) levels I and II. Further research is needed on whether children with bilateral spastic CP and GMFCS levels III, IV, and V show similar results or not.

## 4. Conclusions

To the best of our knowledge, this is the first study to observe the muscle mass of the affected leg with BoNT injection in children with hemiplegic spastic CP using DXA. Our findings indicate that muscle mass on the affected leg decreases at 4 weeks after injection but significantly recovers 12 weeks after injection.

## 5. Study Design and Methods

The study was a prospective cohort study. It is registered as a clinical trial (No. KCT0004591). This study was in accordance with the ethical standards and Guidelines for Good Clinical Practice. All parents of the participants provided written informed consent before their participation.

### 5.1. Participants

We prospectively recruited children aged 2–12 years with spastic hemiplegic CP who presented with spastic equinus foot on the affected side. Patients with Gross Motor Function Classification System (GMFCS) levels I or II without joint contractures were included. Children who had received a BoNT injection within the previous 6 months or who had undergone orthopedic surgery including selective dorsal rhizotomy or intrathecal baclofen treatment were excluded.

### 5.2. Procedures

The muscle(s) selected for injection were determined by functional goals, physical examination, and gait analysis. BoNT dose (Botox^®^; Allergan, Inc., Irvine, CA, USA) was empirically selected for each muscle, and the total dose did not exceed 12 U/kg. The injection site was localized by electrical stimulation and guided by ultrasonography. The injections were administered by physicians who had at least 10 years of experience of injecting BoNT in children with CP.

### 5.3. Assessment of LBM

**Figure 2:**
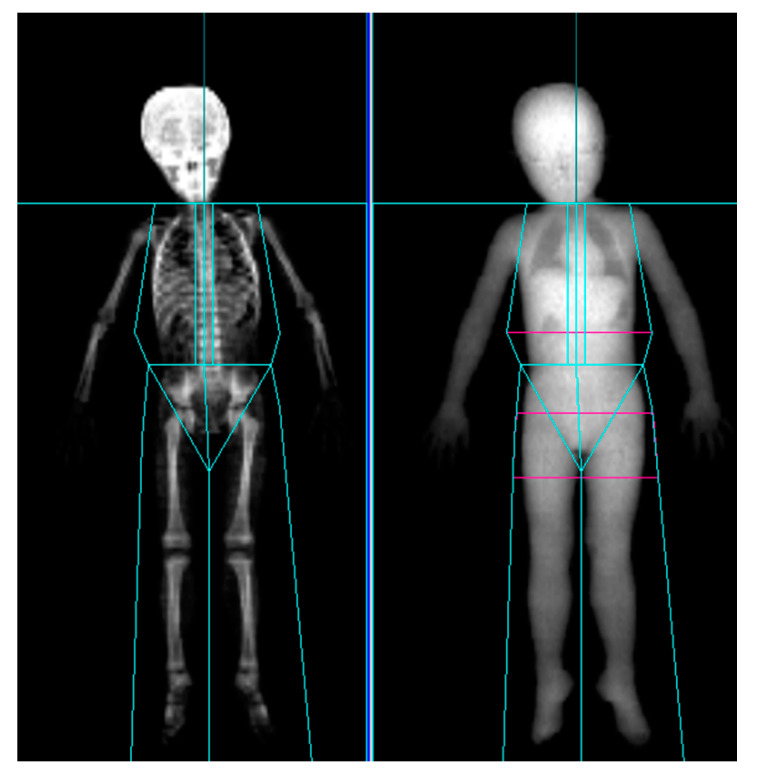
Dual-energy X-ray absorptiometry scan of a 4-year-old cerebral palsy patient showing positions of regional markers.

LBM was measured by DXA preinjection as well as at 4 and 12 weeks after injection. At each acquisition, LBM of the affected leg, unaffected leg, and both upper limbs were collected. All participants were scanned using GE Healthcare Prodigy densitometers (GE Healthcare, Madison, WI, USA) in a routine clinical manner, in accordance with the manufacturer’s recommendations. For Prodigy, Encore version 14.1 software (GE Healthcare) was used for the analysis and acquisition (Figure 2). As anatomical landmarks of the arms and legs, lines perpendicular to the axis of the femoral neck and angled with the pelvic brim and center of the arm socket were drawn. Soft tissue extending from the femoral neck to the toes and from the humeral head to the fingertips were included [25,30].

### 5.4. Statistical Analysis

Statistical analyses were performed using IBM SPSS 23 software (version 23.0, IBM Corporation, Armonk, NY, USA). Repeated measure analysis of variance (ANOVA) test was used to examine the changes in LBM. A *p* value of less than 0.05 was considered as statistically significant.

## Figures and Tables

**Table 1:** Baseline characteristics of the participants.

Characteristic		*n* = 15
Sex (*n*)	male	8
	female	7
Age, mean (SD)		5.27 (2.52)
Body weight (kg), mean (SD)		21.56 (7.47)
GMFCS level (*n*)	I	14
	II	1
Side of hemiplegia (*n*)	Right	9
	Left	6
Previous BoNT injection (*n*)	None	5
	Once	4
	Twice	1
	Three or more times	5
Time from previous BoNT injection	6 month–1 year	5
	1–2 years	1
	2–3 years	2
	Over 3 years	2

GMFCS, Gross Motor Function Classification System; SD, Standard Deviation; BoNT, botulinum toxin.

**Table 2:** Lean body mass of the affected leg, unaffected leg, total arms, and total extremities analyzed by dual-energy X-ray absorptiometry before and after botulinum toxin injection.

Body Part	Preinjection	4 Weeks	12 Weeks
Affected leg (g)	2018 (868)	1982 (820)	2154 (948) ^†^
Unaffected leg (g)	2220 (951)	2288 (948) *	2398 (1011) ^†^
Total arms (g)	1180 (479)	1192 (489)	1260 (520) ^†^
Total extremities (g)	5418 (2287)	5462 (2247)	5811 (2470) ^†^

Data are presented as mean (standard deviation). * *p* < 0.001, significant increase compared with initial lean body mass. ^†^ *p* < 0.005, significant increase compared with preinjection and 4 weeks after injection. g, gram.

**Table 3:** Ratio of lean body mass of the affected and unaffected leg to total body lean body mass (%) after botulinum toxin injection.

Ratio (%)	Preinjection	4 Weeks	12 Weeks
Affected leg/total limbs	37.14 (0.90)	36.22 (1.16) *	36.91 (0.76) ^†^
Unaffected leg/total limbs	40.88 (1.36)	41.83 (1.53) *	41.22 (1.44)

Data are presented as mean (standard deviation). * *p* = 0.001, significant difference compared with the initial lean body mass. ^†^ *p* = 0.05, significant difference compared with lean body mass 4 weeks after injection.

**Table 4:** Ratio of lean body mass of the affected leg to the unaffected leg (%) after botulinum toxin injection.

Ratio	Preinjection	4 Weeks	12 Weeks
affected leg/unaffected leg (%)	90.9 (3.7)	86.8 (4.9) *	89.8 (3.5) ^†^

Data are presented as mean (standard deviation). * *p* < 0.001, significant decrease compared with the initial lean body mass. ^†^ *p* < 0.05, significant increase compared with 4 weeks after injection and no significant difference compared with preinjection.

## Data Availability

Data are contained within the article or supplementary materials.
